# The Human Teeth

**Published:** 1882-07

**Authors:** 


### THE HUMAN TEETH.

Many suppose that the human teeth did not formerly decay as in the present age, and that the dental art is of recent origin; but, in looking over the history of dental surgery, we trace it back more than four hundred years before Christ.

Hippocrates and Herodotus were among the Greek writers upon this subject. And from among the Romans, we have also the writings of Pliny, Martial, Horace, Celsus, and others, which reflect much light upon this branch of surgery; and although as a science it was then in a rude state, yet there were those who devoted their exclusive attention to the teeth at that time. Good gold fillings have recently been found in the teeth of mummies, which must have been inserted more than two thousand years ago.

In fifteen hundred and seventy-nine, Pakie, a celebrated French surgeon, wrote a very correct essay upon the teeth. He enjoyed a great medical reputation, and was appointed surgeon in ordinary to Henry II., which office he held under three succeeding kings.

His cure for the toothache was to thrust a hot wire into the roots, or make an application of the oil of vitriol. He also taught the doctrine of transplanting teeth, which was done by extracting sound teeth from the mouth of one person, and inserting them in that of another of higher rank, whose teeth were decayed or uncomely. In this operation, the decayed teeth are first extracted; immediately after which the sound teeth are removed (generally from the mouth of a servant), and inserted while warm and fresh into the sockets just vacated for their reception. If this operation is well performed, under favorable circumstances the teeth and sockets in a few weeks become united and remain firm for many years. But this method has now become obsolete, and also that of tying artificial teeth with strings or wire.

Artificial teeth made of bone or ivory, were objectionable on account of their unnatural appearance, their liability to rapid decay in the mouth, and consequent tendency to become offensive. These objections led to the introduction of porcelain or incorruptible teeth, which were first conceived by Duchateau in seventeen hundred and seventy four, but not being a dentist, he was unable to carry out his theory practically, although he made some specimens that were capable of being worn, for which the Academy of Medicine of France granted him the honor of a seat.

